# What is the association between gender and self-perceived health status when controlling for disease-specific conditions? A retrospective data analysis of pre- and post-operative EQ-5D-5L differences in total hip and knee arthroplasty

**DOI:** 10.1186/s12891-023-07026-0

**Published:** 2023-11-27

**Authors:** Anja Y. Bischof, Viktoria Steinbeck, David Kuklinski, Carlos J. Marques, Karina Bohlen, Karl C. Westphal, Frank Lampe, Alexander Geissler

**Affiliations:** 1 School of Medicine, Chair of Health Care Management, University of St. Gallen, St. Jakob-Strasse 21, St. Gallen, 9000 Switzerland; 2 Department of Health Care Management, Technical University Berlin, Strasse des 17. Juni 135, Berlin, 10623 Germany; 3 Department of Performance, Neuroscience, Therapy, and Health, Institute of Interdisciplinary Exercise Science and Sports Medicine, Medical School Hamburg, University of Applied Sciences and Medical University, Am Kaiserkai 1, Hamburg, 20457 Germany; 4 Orthopedic and Joint Replacement Department, Schoen Clinic Hamburg Eilbek, Dehnhaide 120, Hamburg, 22081 Germany; 5 Orthopedic and Joint Replacement Department, Schoen Clinic Neustadt, Am Kiebitzberg 10, Neustadt, Holstein 23730 Germany; 6 Orthopaedics, Tumour Orthopaedics & Centre for Endoprosthetics, Asklepios Clinic Barmbek, Rübenkamp 220, Hamburg, 22307 Germany; 7 Faculty of Life Sciences at the Hamburg University of Applied Sciences, Lohbrügger Kirchstraße 65, Hamburg, 21033 Germany

**Keywords:** Total hip arthroplasty, Total knee arthroplasty, Patient-reported outcome measures (PROMs), Gender differences, EQ-5D-5L

## Abstract

**Background:**

The self-perceived health status of patients undergoing total hip and knee arthroplasty (THA and TKA) might differ post-operatively due to gender, age, or comorbidities. Patient-reported outcome measures (PROMs) such as the EQ-5D-5L measure the self-perceived health status. This study investigates whether the index score of the EQ-5D-5L is a valid tool for interpreting gender differences in outcomes for patients undergoing THA and TKA.

**Methods:**

Routine and PROM-data of elective primary THA or TKA patients in two German hospitals between 2016 and 2018 were analyzed. Univariate analysis with Pearson’s chi-square was conducted to identify control variables for gender. To quantify the association between gender and the EQ-5D-5L dimensions, a cumulative odds ordinal logistic regression with proportional odds was conducted.

**Results:**

Two thousand three hundred sixty-eight​​ THA patients (m = 978; f = 1390) and 1629 TKA patients (m = 715; f = 914) were considered. The regression analysis of the individual EQ-5D-5L dimensions showed that female gender was significantly associated with better self-care (THA and TKA) and better post-operative mobility (THA). In contrast, male gender was significantly associated with less pain/discomfort (TKA) and less anxiety/depression (THA) pre-surgery and 3-months post-surgery.

**Conclusion:**

Our results confirmed that the self-perceived health status improved after surgery. However, due to the different associations of gender to the individual dimensions of the EQ-5D-5L, the weighted index score clouds the comparability between patients with different gender undergoing THA or TKA. Therefore, we argue to use the individual five dimensions for health status analysis, to reveal relevant additional information.

**Supplementary Information:**

The online version contains supplementary material available at 10.1186/s12891-023-07026-0.

## Plain Language Summary

Patients undergoing total hip and knee arthroplasty (THA and TKA) can fill out standardized questionnaires pre- and post-surgery, such as the EQ-5D-5L, to measure the improvement in the self-perceived health status. The EQ-5D-5L includes mobility, self-care, usual activity, pain/discomfort, and anxiety/depression. We do not know whether male and female patients experience the same improvement in the dimensions or whether significant differences exist. Currently, only index scores of the EQ-5D-5L are used for the comparison of pre- and post-operative health status. However, due to the questionnaire’s weighted composition, relevant changes in individual dimensions might be easily missed. Thus, we investigated whether significant differences between gender and the EQ-5D-5L dimensions in patients undergoing TKA and TKA are observable. We found that female patients reported significantly better scores in self-care (THA and TKA) and post-operative mobility (THA). In contrast, male gender was significantly associated with less pain/discomfort (TKA) and less anxiety/depression (THA) pre-surgery and 3-months post-surgery. The EQ-5D-5L’s weighted index score, however, does not directly represent these differences. Therefore, we argue to use the individual five dimensions for health status analysis, as relevant additional information on improvement over time would otherwise be missed.

**Supplementary Information:**

The online version contains supplementary material available at 10.1186/s12891-023-07026-0.

## Key points for decision makers

**Table:** 

Patient-reported outcome measures (PROMs), such as the EQ-5D-5L, assess individuals' self-perceived health status. Patients undergoing total hip or knee arthroplasty (THA/TKA) may experience different post-operative self-perceived health statuses influenced by factors like gender, age, or comorbidities. This research aims to examine the validity of using the EQ-5D-5L index score as a tool to interpret gender-related differences in outcomes among THA and TKA patients.
Based on a logistic regression, the study revealed a significant association between female gender and improved self-care (THA and TKA) as well as enhanced post-operative mobility (THA). On the other hand, male gender demonstrated a significant association with reduced pain/discomfort (TKA) and decreased anxiety/depression (THA) both before surgery and at the 3-months post-surgery.
Healthcare professionals and policymakers need to increase their understanding of gender-specific disparities in health status. This is essential to guarantee suitable treatment approaches that address the unique needs of patients. When assessing patients' health, examining the individual dimensions yields more comprehensive, diverse, and practical information compared to relying solely on an index score.

## Background

Patient-reported outcome measures (PROMs) quantify the self-perceived health status of patients. Over the past years, they gained higher utilization in clinical care [1–3] and digital health interventions [4–6]. Two main PROM groups exist: 1) generic such as the EuroQol five-dimension five-level questionnaire (EQ-5D-5L), and 2) disease-specific such as the Western Ontario and McMaster Universities Osteoarthritis questionnaire (WOMAC), focusing on hip and knee joints [7]. Disease-specific instruments complement generic instruments, as they highlight disease-specific problems regarding a particular disease that a generic instrument cannot cover. However, both instruments allow for objectively evaluating patients’ health status in a valid, reliable, and systematic manner [8–10].

Total hip and knee arthroplasty (THA and TKA) are standardized treatments. Female patients are up to three times more likely to undergo TKA at a more advanced stage [11–13]. Multiple studies reported that the pre-operative [13–15] and sometimes also post-operative self-perceived health status [16] differ by gender. The reasons for these differences are assumed to be diverse. Literature suggests various reasons for female patients experiencing worse health status such as being referred to a surgeon with a higher degree of disability, higher risk of complications, and unwillingness to accept surgery [12, 14, 17]. The unwillingness to accept surgery might be developed due to a general increase in the perceived risk for morbidity and mortality after surgery or previous negative experiences in health systems due to gender biases [18–20]. Additionally, female patients tend to be older when undergoing arthroplasty, especially in case of THA [17, 21], and age is negatively associated with health status [22–25]. Regarding EQ-5D-5L scores, female gender, older age, and low sociodemographic status are related to worse scores in most dimensions in the general population [22, 26, 27]. Furthermore, differences in (total) scores by gender are also observed in the WOMAC, as female patients indicated more problems especially with pain. Therefore, the literature emphasizes evaluating and elaborating more gender-specific treatment strategies [28–30].

To enhance gender-specific treatment strategies, previous research highlighted the consideration of the individual dimensions of the EQ-5D-5L instead of the index score as the deconstruction provides insights into the contribution of the individual dimensions to the index score [26]. Additionally, the index score of the EQ-5D-5L is established based on national preferential weights, where cross-society preferences are reflected. The aim of selecting respondents for the national weighing is to achieve a quota-based gender ratio according to national statistics. The German value set was established on a participant dataset consisting of 53.4% female respondents [31]. Nevertheless, cross-society preference weights do not have to reflect specific preferences of individual sub-groups, such as patients undergoing THA or TKA. Therefore, analyzing dimensional scores becomes critical in stratifying and understanding differences in patients’ health status composition.

While there is research on health status differences by gender [14, 32, 33], it has not been analyzed whether systematic gender-specific differences are observable in the dimensions of the EQ-5D-5L for THA and TKA patients pre- and post-surgery. A granular analysis of the differences per dimension helps to understand patients’ gender-specific health status pre- and post-surgery, and their recovery pathway. Consequently, these findings can be used to improve outcomes from THA and TKA, by e.g., more accurately monitoring recovery pathways and adapting treatment protocols according to health status development. To the best of our knowledge, no study has yet assessed THA and TKA patients’ values of EQ-5D-5L dimensions by gender over time. Hence, the research question is: *“What are the differences in the association between gender and the EQ-5D-5L dimensions in patients undergoing total hip and knee arthroplasty pre- and post-surgery?”.*

## Methods

### Study design

This study is a retrospective data analysis based on a prospectively collected dataset. The STROBE Statement guidelines for reporting observational studies were followed [34]. The Ethic-Commission of the Federal State of Hamburg, Germany, was informed and approved the study (2021–300010-WF). Data was saved, processed, and analyzed anonymously.

### Patients

**Fig. 1:**
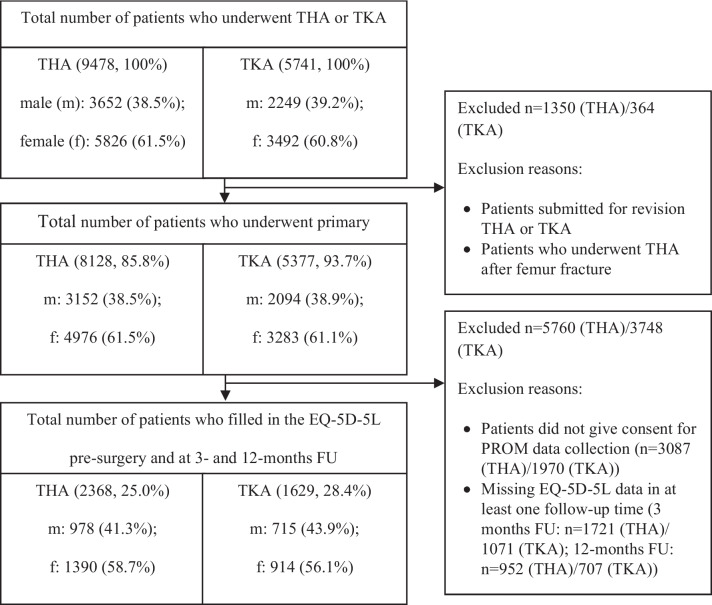
Patient flow diagram by follow-up time, procedure, gender and EQ-5D-5L response Legend: FU: Follow-up; THA: Total Hip Arthroplasty; TKA: Total Knee Arthroplasty. The percentages after the total number of THA and TKA patients indicate the retention rate. The percentages after male and female gender indicate the distribution within the remaining sample

PROM- and routine data of 2368 patients (male (m) = 978; female (f) = 1390) undergoing elective primary THA and 1629 patients (m = 715; f = 914) undergoing elective primary TKA in two private German hospitals belonging to the same hospital chain between January 2016 and December 2018 were analyzed. PROM-data is not routinely collected in German hospitals. Therefore, the patients were asked to give their written consent. Patients filled in the pre-surgery (at the day of admission), 3- and 12-months post-surgery EQ-5D-5L, and WOMAC questionnaires. The data of patients who underwent revisional THA or TKA, or THA after femur fracture and those who did not consent to PROM-data collection were excluded from the analysis (Fig. 1).

### Patient-reported outcome instruments

The EQ-5D-5L encloses the following five dimensions: mobility, self-care, usual activities, pain/discomfort, and anxiety/depression. Each dimension provides five answer levels: no, mild, moderate, severe, and extreme or unable to [35]. The EQ-5D-5L health index scores (also called *values*) ranges from 0 (death) to 1 (perfect health). It might even drop into a negative range, where certain health status is considered worse than death. EQ-5D-5L values are calculated based on national preference weights defined per dimension – called value sets [8, 36]. The answers on each dimension are used to calculate the index score according to national preference weights. In the German value set, pain/discomfort and anxiety/depression are allocated with higher utility weights than the functional dimensions: mobility, self-care, and usual activities. Additionally, the dimension pain/discomfort has the highest impact on the health status [31]. The latest EQ-5D version with five levels was chosen due to its improved informational value, higher sensitivity, and accuracy in health status measurement [37, 38].

The WOMAC – a disease-specific instrument for the evaluation of hip and knee osteoarthritis [9, 10] – includes 24 items covering three dimensions – pain (5 items), stiffness (2 items), and physical function (17 items). Each dimension of the WOMAC consists of five levels: none, mild, moderate, severe, or extreme. Within a dimension, the scores of all items are added up. This results in pain taking values from 0 to 20, stiffness oscillates between 0 to 8, and physical function ranges from 0 to 68. The total WOMAC score is generated by summing up all values of the individual dimensions. The lower the total or individual-dimension WOMAC score, the less the patient suffers from pain, stiffness, or physical limitations [39, 40].

### Data analysis

We used the PROMs (EQ-5D-5L, WOMAC) and routine hospital data on patient characteristics (such as sociodemographic data, medical conditions, and surgical details) from THA and TKA patients operated at two German hospitals. PROMs were measured pre-surgery, 3-months, and 12-months post-surgery. The dimensions of the EQ-5D-5L serve as main area of investigation.

Differences regarding the surgical procedures and gender were described by independent-samples t-test or Mann–Whitney U depending on the variable’s characteristics or distribution. The health state indices of the EQ-5D-5L were calculated based on the German value set [31].

To quantify the association between gender and the EQ-5D-5L dimensions, a cumulative odds ordinal logistic regression with proportional odds was conducted [25] (for the formula and additional information see Online Resource 1). Age was included as control variable in all calculations. The relationship between age (in years) and health status has been extensively discussed [22, 26, 41, 42]. Other control variables such as the Elixhauser Comorbidity Index, walking distance, and walking aid pre-surgery, or surgery duration were included in the calculations based on the correlation significance of Pearson’s chi-square between gender and the control variables (for results see Online Resources 2 and 3).

At each measuring point (pre-, 3-, and 12-months post-surgery) the corresponding WOMAC total score was included as a control variable for each patient. Literature provides evidence that the WOMAC subscale scores – especially pain and functional disability – are associated with changes in the EQ-5D-5L values, i.e., disease-specific PROMs are interrelated with generic health states [43]. However, since the WOMAC total score is built of the sum of its subscale scores – and no weighing of individual subscales applies – the total score was considered in the ordinal logistic regression.

All analyses and calculations were performed separately for patients who underwent THA and TKA to quantify the association between gender and health status more precisely. All analyses were conducted with IBM-SPSS version 28 [44].

## Results

### Descriptive statistics

The average age of male THA patients was 68.72 years (f = 68.9 years), with a mean length of hospital stay of 7.34 days (f = 7.46 days) and a mean surgery time of 46.47 min (f = 45.76 min). The specific and general in-hospital complication ratios were around 2% or lower for both gender. Regarding the WOMAC total scores, male patients started with lower values, i.e., better scores. The scores of the male patients remained lower (better) 12-months post-surgery compared to female patients. However, both genders experienced an improvement in total WOMAC scores over time, while significant score differences between the gender existed preoperatively (*p* < 0.001) and at 3-months post-surgery (*p* = 0.019) (Table 1).

**Table 1:** Descriptive statistics

Variable	THA		TKA	
	**Male patients** (*n* = 978)	**Female patients** (*n* = 1390)	**Male patients** (*n* = 715)	**Female patients** (*n* = 915)
Age (years)	68.72 (9.05)	68.86 (9.18)	71.00^**^ (7.90)	70.00^**^ (8.70)
LOS (days)	7.34 (4.16)	7.43 (4.01)	7.44 (3.55)	7.37 (3.42)
Surgery duration (min.)	46.47 (18.06)	45.76 (17.05)	57.83^***^ (17.67)	55.26^***^ (18.72)
In-hospital complication ratio (general)	0.03 (0.16)	0.02 (0.14)	0.03 (0.16)	0.02 (0.12)
In-hospital complication ratio (specific)	0.01 (0.10)	0.01 (0.11)	0.02^**^ (0.12)	0.04^**^ (0.07)
EQ-5D-5L (pre-surgery)	0.576^***^ (0.275)	0.512^***^ (0.291)	0.625^***^ (0.253)	0.534^***^ (0.284)
EQ-5D-5L (3-month FU)	0.883^**^ (0.156)	0.867^**^ (0.172)	0.844^***^ (0.163)	0.817^***^ (0.182)
EQ-5D-5L (12-month FU)	0.910 (0.149)	0.903 (0.160)	0.885^***^ (0.154)	0.862^***^ (0.169)
WOMAC (pre-surgery)	45.66^***^ (15.52)	50.15^***^ (14.61)	41.77^***^ (15.28)	46.44^***^ (14.39)
WOMAC (3-month FU)	13.05 (12.19)	14.36 (12.59)	17.87^***^ (13.45)	20.00^***^ (14.28)
WOMAC (12-month FU)	11.02 (12.98)	11.98 (13.89)	14.43^***^ (13.64)	17.54^***^ (15.13)
Elixhauser Comorbidity Score	3.80^**^ (4.96)	3.31^**^ (4.55)	3.46 (4.77)	3.26 (4.34)

The following in-hospital complications are categorized as specific: Misplacement of the implant; secondary dislocation of the implant; closed or open reposition of the implant; lesion of vessels; lesion of nerve roots; periprosthetic fractures and wound infections. General in-hospital complications are defined as: Pneumonia; urinary tract infection; sepsis; pulmonary embolism; venous thromboembolism; cardiac complications; etc. For reasons of simplicity and clarity, the most informative variables are shown. A full table of all variables used in the further analysis is attached to Online Resource 4

Statistically significant differences between gender are indicated by *p* < 0.05^**^ and *p* < 0.01^***^

*LOS* Length of stay, *SD* Standard Deviation

On average, male TKA patients were 71.0 years old, whereas female patients were one year younger at surgery (*p* = 0.015). After TKA, male patients stayed on average 7.44 days (f = 7.37 days) in the hospital, and the mean surgery time was 57.83 min (f = 55.26 min, *p* = 0.005). When referring to the in-hospital complication ratio, a significant difference between gender was observable only for specific complications. The WOMAC scores for male patients were lower pre- and post-surgery compared to female patients. However, both gender showed WOMAC score improvements over time, while the scores between the gender remained significantly different over all measuring points (Table 1).

### Gender differences in generic health index score (EQ-5D-5L) over time

Male patients in the THA group perceived a mean health index score improvement of 0.334 between pre-operative and 12-months post-surgery, while female patients experienced an improvement of 0.391. Hence, female patients perceived a larger health status improvement within the first 12 months after surgery (*p* < 0.001).

**Fig. 2:**
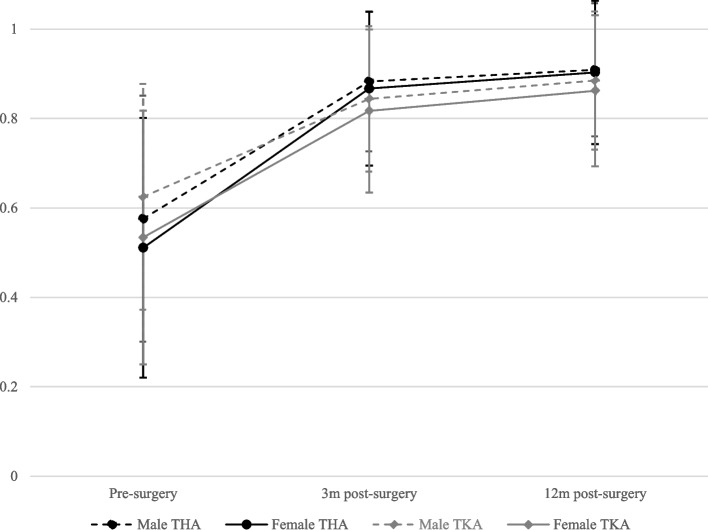
Mean EQ-5D-5L total score and corresponding standard deviations for the patients in the total hip and total knee arthroplasty groups by gender over time Legend: THA: Total Hip Arthroplasty; TKA: Total Knee Arthroplasty

In the TKA group, male patients had on average a pre-operative health status index of 0.625 which improved to 0.885, while female patients showed an improvement from 0.534 to 0.862. Thus, female patients started with lower values, and despite higher improvement rates, they did not experience the same post-operative health status enhancement as male patients. However, the improvement rates between gender tend to be significantly different (*p* < 0.001) (Fig. 2).

When comparing the two types of arthroplasties, TKA patients started with higher EQ-5D-5L total scores (m = 0.625, f = 0.534) compared to THA patients (m = 0.576, f = 0.511). However, THA patients showed higher mean health status values 3-months post-surgery (THA: m = 0.883, f = 0.867; TKA: m = 0.844, f = 0.817). At 12-months post-surgery, all patient groups recorded a health status improvement over time. Nevertheless, THA patients showed a larger overall health status improvement (THA: m =  + 0.334, f =  + 0.391; TKA: m =  + 0.260, f =  + 0.328).

### Comparison of EQ-5D-5L dimension scores by gender

In the THA group, significant differences regarding gender were observed in the dimensions of self-care and anxiety/depression at all measuring points. Additionally, significant differences were detected in usual activity and pain/discomfort pre-surgery. However, this effect was significant only for pain/discomfort 3-months post-surgery. In the TKA group, the dimensions of pain/discomfort and anxiety/depression differed significantly per gender pre- and post-surgery. Furthermore, mobility and usual activity differed significantly concerning gender, but this effect was no longer present post-surgery (Table 2). For both THA and TKA, the dimension with the lowest scores was pain/discomfort pre-and post-surgery. Furthermore, both patient groups indicated the least problems in the dimension self-care pre- and post-surgery, while in THA usual activity reached the same level as self-care 12-months post-surgery.

**Table 2:** Comparison of the EQ-5D-5L dimensions by gender

	**THA**		**TKA**	
	**Male Mean (SD)**	**Female Mean (SD)**	**Male Mean (SD)**	**Female Mean (SD)**
Mobility				
Pre-surgery	1.81 (0.73)	1.86 (0.72)	1.75^***^ (0.72)	1.82^***^ (0.67)
FU3	1.32 (0.56)	1.30 (0.57)	1.34 (0.57)	1.34 (0.57)
FU12	1.37 (0.71)	1.31 (0.64)	1.37 (0.63)	1.42 (0.72)
Self-care				
Pre-surgery	1.27^***^ (0.55)	1.21^***^ (0.50)	1.15 (0.42)	1.13 (0.42)
FU3	1.08^**^ (0.31)	1.06^**^ (0.28)	1.08^**^ (0.28)	1.06^**^ (0.30)
FU12	1.11^***^ (0.39)	1.07^***^ (0.33)	1.13 (0.44)	1.11 (0.41)
Usual activity				
Pre-surgery	1.66^***^ (0.73)	1.73^***^ (0.73)	1.55^***^ (0.69)	1.66^***^ (0.68)
FU3	1.25 (0.51)	1.27 (0.51)	1.32 (0.55)	1.34 (0.56)
FU12	1.11^***^ (0.39)	1.07^***^ (0.33)	1.32^***^ (0.60)	1.42^***^ (0.69)
Pain/discomfort				
Pre-surgery	2.34^***^ (0.65)	2.47^***^ (0.66)	2.24^***^ (0.64)	2.41^***^ (0.66)
FU3	1.44^***^ (0.57)	1.51^***^ (0.61)	1.63^***^ (0.59)	1.74^***^ (0.60)
FU12	1.43 (0.65)	1.46 (0.67)	1.55^***^ (0.67)	1.73^***^ (0.77)
Anxiety/depression				
Pre-surgery	1.28^***^ (0.55)	1.47^***^ (0.65)	1.18^***^ (0.42)	1.46^***^ (0.67)
FU3	1.08^***^ (0.30)	1.14^***^ (0.39)	1.11^***^ (0.37)	1.17^***^ (0.42)
FU12	1.14^***^ (0.45)	1.18^***^ (0.50)	1.13^***^ (0.43)	1.19^***^ (0.50)

Statistically significant difference between gender at a ^**^95% and ^***^99% significance level

*SD* Standard Deviation,* FU* Follow-up

### Association of gender with the EQ-5D-5L dimensions

**Fig. 3:**
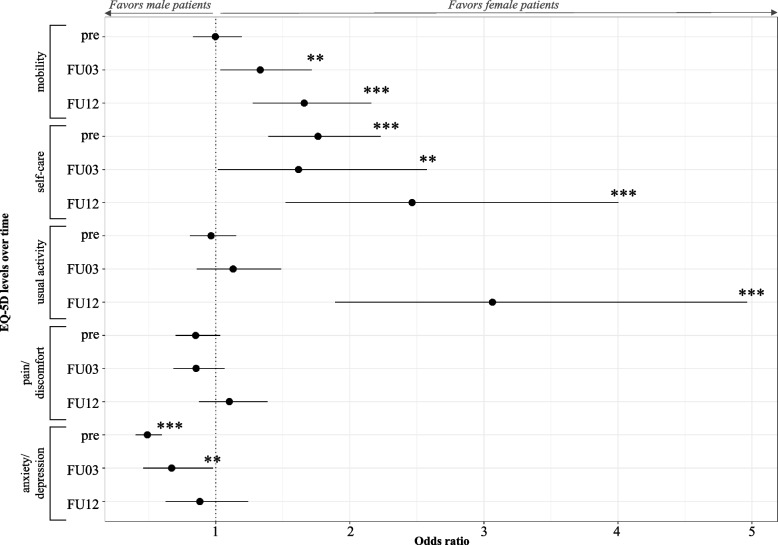
Association of gender with EQ-5D-5L dimensions of total hip arthroplasty over time Legend: Values are odds ratio and their 95% CI for female gender; significant Odds ratio at a **95% and ***99% significance level, FU = Follow-up; Notes: Coefficients of the control variables related to age, Elixhauser Comorbidity Index, WOMAC, pain, the modified Kellgren-Lawrence Classification, severity of joint destruction in rheumatic diseases, pre-operative conditions, walking distance and aid at admission were included in the pre-surgical dimensions, while early mobilization, clinic type, length of stay, and surgery duration were added to the post-operative regressions, too. Additionally, pre-surgical EQ-5D-5L dimensions were included in the post-operative regressions to avoid omitting the effect of pre-surgical conditions on post-surgical outcomes. The coefficients of the control variables are attached to Online Resource 5

When controlling for confounders such as age, pre-surgical conditions, and disease-specific health status in the ordinal logistic regression, the association of gender with the individual EQ-5D-5L dimensions was investigated. In the THA group, female patients showed significantly higher odds for better (lower) mobility levels post-surgery, although pre-surgery the odds ratio was slightly, but non-significantly, in favor of male gender (OR = 0.996). In the dimension of self-care, female gender was significantly associated with better self-care levels at baseline and 3- and 12-month follow-ups. In the dimension of usual activities, female patients achieved better scores post-surgery. However, the association is significant only 12-months post-surgery. In contrast, female gender was significantly associated with worse values (decreased odds) in the dimension anxiety/depression pre- and 3-month post-surgery. Nevertheless, this association was no longer significant 12-months post-surgery. Furthermore, in pain/discomfort, there were no significant associations with either gender, while the odds ratio favored male gender in the first two measuring points, 12-months post-surgery female gender was associated with better scores (Fig. 3).

**Fig. 4:**
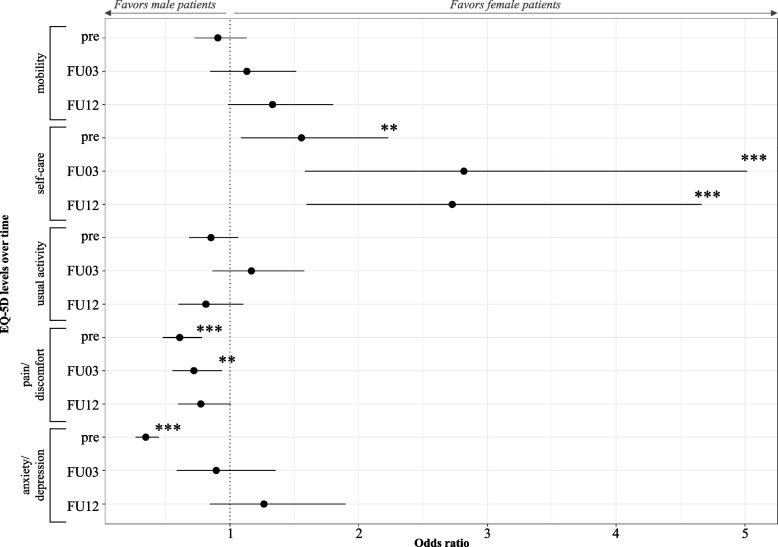
Association of gender with EQ-5D-5L dimensions of total knee arthroplasty over time Legend: Values are odds ratio and their 95% CI for female gender; significant Odds ratio at a **95% and ***99% significance level, FU = Follow-up; Notes: Confidence intervals are set to 95%. For TKA, coefficients of the control variables related to age, Elixhauser Comorbidity Index, WOMAC, pain, severity of joint destruction in rheumatic diseases, pre-operative findings, patient clinical complexity level (PCCL, a measure for the degree comorbidities), walking distance and aid at admission were included in the pre-surgical dimensions, while post-operative condition, surgery duration, walking distance and aid at discharge were added to the post-operative regressions, too. Additionally, pre-surgical EQ-5D-5L dimensions were included in the post-operative regressions to avoid omitting the effect of pre-surgical conditions on post-surgical outcomes. The coefficients of the control variables are attached to Online Resource 6

The pre-surgical mobility odds ratio is slightly better for male gender (OR = 0.906), whereas female patients tend to display better scores post-surgery. However, none of the associations for mobility are significant. Female gender is significantly associated with better scores in self-care pre- and post-surgery. No clear association to one of the gender is observable in usual activity. In contrast, being a male patient is significantly associated with better scores in pain/discomfort pre- and 3-month post-surgery. Additionally, being of female gender was significantly associated with worse scores in the dimension anxiety/depression preoperatively. However, this effect is no longer observable post-surgery (Fig. 4).

## Discussion

This paper comprehensively investigated the relationship between gender and the dimensions of the EQ-5D-5L. Our results confirmed previous studies on the potential of THA and TKA in improving quality of life since self-perceived health status improved significantly after surgery [17, 21, 42, 45]. From a gender perspective, female patients experienced higher health status improvements in total scores than male patients, however, remained at a lower level in the total scores post-surgery. This was measured with both PROM instruments (i.e., the EQ-5D-5L and WOMAC). The results are in line with previous studies highlighting this imbalance, especially pre-surgery [13, 15, 26]. The pre-surgical health condition is the strongest predictor of health gains after surgery [42, 46].

Diving into the dimensions of the EQ-5D-5L, male patients rated most of the dimensions with lower, i.e., more favorable, levels. Male gender was significantly related to better (lower) anxiety/depression (THA) and pain/discomfort (TKA) levels pre- and 3-month post-surgery. This effect diminished 12-month post-surgery. Interestingly, Casetta et al. [47] found that the mental health state of female patients remained better while facing chronic conditions such as multiple sclerosis, although physical functioning decreased. Contrarily, our conclusions lead to the opposite assumption of male patients showing better mental health (anxiety/depression) pre- and post- arthroplasty. Potential explanations could be the underreporting of mental health concerns among male patients or female patients’ increased pain sensitivity in combination with a certain degree of severity of the chronic disease leading to more mental health constraints [15, 48]. This argument is also supported by the significant association of lower pain/discomfort scores with male gender in our study.

The different associations of gender with the individual dimensions of the EQ-5D-5L indicate that the outcomes of THA and TKA need to be considered and interpreted separately. Self-care seems to be the dimension that is significantly positively associated with female gender at all measuring points, independent of either undergoing THA or TKA. However, male patients show significant positive associations to dimensions with higher weights in the EQ-5D-5L – anxiety/depression (THA) and pain/discomfort (TKA).

In many national EQ-5D-5L value sets, like in the German value set, more weight is set on the dimension anxiety/depression and pain/discomfort compared to self-care [31]. The weighted preferences determine how EQ-5D-5L value sets are calculated and used in decision-making processes [8]. Therefore, this aspect leads to the discussion of whether the different perceptions of well-being of female THA or TKA patients are sufficiently weighted when using the EQ-5D-5L as index score or whether an analysis per dimension creates a more accurate picture of gender-specific health status.

In the investigated patient population, female TKA patients were younger than their male counterparts. Former studies differed in their statements of whether male or female TKA patients were older at the point of surgery, but at times referred to the age as explanatory variable for gender difference [14, 18, 49]. This relationship could not be confirmed in our analysis. Contrarily, no significant difference in frequency of in-hospital complications between male and female patients was measurable, which confirms previous findings [13, 50].

Our results show the need to put more emphasis on the individual dimensions of PROMs when interpreting the outcomes concerning gender differences, especially regarding the rising demand in clinical practice and digital health interventions. The relationship of gender with mobility (THA), self-care (THA and TKA), anxiety/depression (THA), and pain/discomfort (TKA) were significant when controlling for other relevant variables. Although female patients tended to show better scores in self-care over all measuring points, they only achieved lower index scores due to the weighing of the individual dimensions of the EQ-5D-5L in the German value set. This might falsify the meaning of the index score that female patients were worse off pre- and post-surgery, as they tend to indicate better scores in dimensions that are associated with lower utility weights.

Our findings suggest that using a single index score for evaluating patients' health status without gender consideration is questionable. An alternative strategy to enhance the comparability of results would be the implementation of gender-specific index scores or a greater reliance on the distinct dimensions of the EQ-5D-5L, some of which exhibit gender-related associations. This approach may additionally facilitate the operational utility of PROMs, such as in monitoring treatment regimens or delineating recovery trajectories, thus enabling informed decision-making about treatment adaptations. To promote the broader integration of PROMs within clinical practice, it is essential to establish comprehensive guidelines for accurately assessing PROMs and the subsequent derivations that can be drawn from them.

### Limitations

As stated above, this is a retrospective data analysis. We used routine- and PROM-data that was collected for quality control purposes. We identified three limiting factors of this research: First, missing profile items in the EQ-5D-5L led to the exclusion of the corresponding patients, as substituting an average derived from other non-missing items for missing profile items is considered bad practice, and the individual dimensions serve as dependent variables in the conducted regressions. Second, we included all patients in our sample independent of comorbidities due to the dataset's lack of information. The number and degree of comorbidities might influence the patient's perception of the health status and, thus, change the EQ-5D-5L index score. Third, there are also limitations to the generalization of results from this dataset since the data originates from two health care providers belonging to the same provider network. Data collection on information such as previous treatment approaches or current living situation are not part of the routine data collection in Germany. These additional variables would have allowed for more precise interpretation of the relationship between gender and post-surgery outcomes.

Future research should apply the presented methodological approach to comparable datasets from other countries. If similar findings result, it should be investigated whether gender-specific adjustments of the EQ-5D-5L are necessary to ensure comparability of results between gender in people undergoing THA or TKA. In addition, including a longer than 12-months post-surgery follow-up period might further improve the understanding of the relationship between gender and the outcome parameters in the long-term.

## Conclusion

Gender was significantly related to individual dimensions of the EQ-5D-5L in the THA and TKA groups, pre- and post-surgery when controlling for age, comorbidity, disease-specific health status, and pre-surgical conditions. Male THA and TKA patients perceived significantly better levels than female patients for most dimensions of the EQ-5D-5L. Male patients in the THA group showed significant associations with lower anxiety/depression levels pre- and 3-months post-surgery, while the same holds for pain/discomfort in TKA. Female gender was significantly associated with better mobility post-surgery for THA and better self-care independent of the measuring point for both treatments. Therefore, the awareness of gender-specific differences in health status should inform healthcare professionals and policymakers in integrating these insights into healthcare to ensure appropriate treatment measures based on patients’ needs. When evaluating patients’ health status, considering the individual dimensions reveals more detailed, differentiated, and actionable insights than the index score.

### Supplementary Information

**Online resource 1.** Formula on applied cumulative odds ordinal logistic regression with proportional odds

**Online resource 2.** Correlations of potential control variables with gender for THA.

**Online resource 3.** Correlations of potential control variables with gender for TKA.

**Online resource 4.** Descriptive statistics (full table).

**Online resource 5.** Full regression table for total hip arthroplasty (THA).

**Online resource 6.** Full regression table for total knee arthroplasty (TKA).

## Data Availability

The data used in the present study was collected at the Schoen Clinic Hamburg and at the Schoen Clinic Neustadt, two clinics of the Schoen Clinic Group. The data is property of each clinic and for reasons of protection of data privacy the raw data cannot be made available publicly. The SPSS data files, and SPSS outputs can be provided by the corresponding author upon reasonable request after permission of both clinics.

## Abbreviations

ASA: American Society of Anesthesiologists
EQ-5D-5L: EuroQol five-dimensions five-levels questionnaire
f: Female
m: Male
THA: Total hip arthroplasty
TKA: Total knee arthroplasty
PROMs: Patient-reported outcome measures
WOMAC: Western Ontario and McMaster Universities Osteoarthritis questionnaire
