# Supplementary material for: What is the association between gender and self-perceived health status when controlling for disease-specific conditions? A retrospective data analysis of pre- and post-operative EQ-5D-5L differences in total hip and knee arthroplasty
Source: BMC Musculoskelet Disord. 2023 Nov 27;24:914. doi: 10.1186/s12891-023-07026-0 (PMC10680301; doi:10.1186/s12891-023-07026-0)
Supplement: Supplementary file 1 — Online resource 1. Formula on applied cumulative odds ordinal logistic regression with proportional odds [file 12891_2023_7026_MOESM1_ESM.pdf]

Article title: What is the association between gender and self-perceived health status when controlling for disease-specific conditions? A retrospective data analysis of pre- and post-operative EQ-5D-5L differences in total hip and knee arthroplasty
Journal name: BMC Musculoskeletal Disorders
Author names: Anja Y. Bischof, Viktoria Steinbeck, David Kuklinski, Carlos J. Marques, Karina Bohlen, Karl C. Westphal, Frank Lampe, Alexander Geissler
Corresponding Author: Anja Y. Bischof, M.A., University of St. Gallen, School of Medicine, Chair of Health Care Management, St. Jakob-Strasse 21, 9000 St. Gallen, Switzerland, anja.bischof@unisg.ch

Online Resource 1. Formula on applied cumulative odds ordinal logistic regression with proportional odds

The following formula was used:

$$logit\ [\pi(EQdimension_{time} \leq j \mid X)]$$
$$= \ln\left(\frac{\pi\ (EQdimension_{time} \leq j \mid X)}{\pi\ (EQdimension_{time} \geq j \mid X)}\right)$$
$$= \alpha_j + (-\beta X),$$

where α_j's are the thresholds, β are logit coefficients, and X represents the independent variables. When interpreting the binary variable sex (m/f) and its relation to the ordinal explanatory variables, female sex functions as the reference category. The interpretational logic holds that an odds ratio lower than 1 indicates that being female is associated with worse values in that dimension. However, if the expected β is larger than 1, being of female sex is associated with increased odds for better values in that dimension (1,2). The closer the value gets to 1, the smaller the difference between the sex-specific dimensional scores.

EQdimension stands for the individual dimensions of the EQ-5D-5L instrument (*mobility, self-care, usual activity, pain/discomfort, and anxiety/depression*), which are time-dependent (*pre-surgery*, *3-months post-surgery* and *12-months post-surgery)*. Furthermore, j = 1,2,3,4,5 represent the response levels of the EQ-5D-5L – where 1 indicates "no problems" and 5 represents "extreme problems" in the corresponding dimensions. We used *SPSS PLUM (Polytomous Universal Model)*, an extension of the generalized linear model for ordinal response data (1).

References

1. Liu X. Ordinal regression analysis: Fitting the proportional odds model using Stata, SAS and SPSS. Journal of Modern Applied Statistical Methods. 2009 Nov 1;8(2):632–42.

2. Janssen B, Szende A, Ramos-Goñi JM. Data and methods. In: Szende A, Janssen B, Cabasés J, editors. Self-Reported Population Health: An International Perspective based on EQ-5D. Dordrecht: Springer Netherlands; 2014. p. 7–17.
