# Supplementary material for: What is the association between gender and self-perceived health status when controlling for disease-specific conditions? A retrospective data analysis of pre- and post-operative EQ-5D-5L differences in total hip and knee arthroplasty
Source: BMC Musculoskelet Disord. 2023 Nov 27;24:914. doi: 10.1186/s12891-023-07026-0 (PMC10680301; doi:10.1186/s12891-023-07026-0)
Supplement: Supplementary file 2 — Online resource 2. Correlations of potential control variables with gender for THA. [file 12891_2023_7026_MOESM2_ESM.pdf]

Article title: What is the association between gender and self-perceived health status when controlling for disease-specific conditions? A retrospective data analysis of pre- and post-operative EQ-5D-5L differences in total hip and knee arthroplasty
Journal name: BMC Musculoskeletal Disorders
Author names: Anja Y. Bischof, Viktoria Steinbeck, David Kuklinski, Carlos J. Marques, Karina Bohlen, Karl C. Westphal, Frank Lampe, Alexander Geissler
Corresponding Author: Anja Y. Bischof, M.A., University of St. Gallen, School of Medicine, Chair of Health Care Management, St. Jakob-Strasse 21, 9000 St. Gallen, Switzerland, anja.bischof@unisg.ch

Online Resource 2 Correlations of potential control variables with sex for THA

THA		
**Variable**	**Pearson's chi-square**	**Cramer-V**
Pain (no, stress pain, or rest pain)	24.847***	0.102
**Modified Kellgren-Lawrence Classification**		
Osteophytes	2.078	
Joint space	10.774**	0.067
Sclerosis	8.529**	0.060
Deformation	7.541**	0.056
**Severity of joint destruction in rheumatic diseases**		
Rheumatic disease with manifestation at the affected joint	11.093***	0.068
Erosive joint destruction (according to Larsen-Dale-Eek)	6.008	
**Pre-operative findings**		
ASA classification	24.401***	0.102
Post-operative specific complications	0.485	
Post-operative generic complications	0.946	
Walking distance at admission	9.492**	0.063
Walking aid at admission	13.372***	0.075
Walking distance at discharge	6.156	
Walking aid at discharge	5.199	
Patient Clincial Complexity Level	4.605	
Early mobilization	4.807**	0.047
Clinic type (general vs. specialized)	4.608**	0.044
**Variable**	**Chi-Square**	**Eta**
WOMAC (pre-surgery)	422.109	
WOMAC (3-month FU)	290.700	
WOMAC (12-month FU)	232.479	
Elixhauser Comorbidity Score	67.111***	0.051
Length of stay	76.660***	0.018

Surgery duration	124.728***	0.020
Age	49.334	

Statistically significant difference between sex at a **95% and ***99% significance level; FU=Follow-up. Notes: Pearson's chi-square indicates whether there is a significant correlation to a variable with sex, and Cramer-V or Eta shows the effect size. If there is no significant correlation for a variable, the effect size is not indicated.
