# Supplementary material for: What is the association between gender and self-perceived health status when controlling for disease-specific conditions? A retrospective data analysis of pre- and post-operative EQ-5D-5L differences in total hip and knee arthroplasty
Source: BMC Musculoskelet Disord. 2023 Nov 27;24:914. doi: 10.1186/s12891-023-07026-0 (PMC10680301; doi:10.1186/s12891-023-07026-0)
Supplement: Supplementary file 3 — Online resource 3. Correlations of potential control variables with gender for TKA. [file 12891_2023_7026_MOESM3_ESM.pdf]

Article title: What is the association between gender and self-perceived health status when controlling for disease-specific conditions? A retrospective data analysis of pre- and post-operative EQ-5D-5L differences in total hip and knee arthroplasty
Journal name: BMC Musculoskeletal Disorders
Author names: Anja Y. Bischof, Viktoria Steinbeck, David Kuklinski, Carlos J. Marques, Karina Bohlen, Karl C. Westphal, Frank Lampe, Alexander Geissler
Corresponding Author: Anja Y. Bischof, M.A., University of St. Gallen, School of Medicine, Chair of Health Care Management, St. Jakob-Strasse 21, 9000 St. Gallen, Switzerland, anja.bischof@unisg.ch

Online Resource 3 Correlations of potential control variables with sex for TKA

**TKA**		
**Variable**	**Pearson's chi-square**	**Cramer-V**
Pain (no, stress pain, or rest pain)	28.808***	0.133
**Modified Kellgren-Lawrence Classification**		
Osteophytes	0.268	
Joint space	2.426	
Sclerosis	1.586	
Deformation	1.155	
**Severity of joint destruction in rheumatic diseases**		
Rheumatic disease with manifestation at the affected joint	7.637***	0.068
Erosive joint destruction (according to Larsen-Dale-Eek)	1.732	
**Pre-operative findings**		
Gonarthrosis	6.761**	0.064
Malposition of the knee	96.257***	0.243
ASA classification	32.674***	0.142
Post-operative specific complications	5.329**	0.057
Post-operative generic complications	2.024	
Walking distance at admission	8.724	
Walking aid at admission	11.183**	0.083
Walking distance at discharge	8.537**	0.072
Walking aid at discharge	10.043***	0.079
Patient Clincial Complexity Level	14.029***	0.093
Early mobilization	0.271	
Clinic type (general vs. specialized)	0.633	
**Variable**	**Chi-Square**	**Eta**
WOMAC (pre-surgery)	343.458	
WOMAC (3-month FU)	239.62	
WOMAC (12-month FU)	216.115	

Elixhauser Comorbidity Score	69.557***	0.022
Length of stay	36.295	
Surgery duration	139.742***	0.07
Age	65.554	

Statistically significant difference between sex at a **95% and ***99% significance level; FU=Follow-up. Notes: Pearson's chi-square indicates whether there is a significant correlation to a variable with sex, and Cramer-V or Eta shows the effect size. If there is no significant correlation for a variable, the effect size is not indicated.
