# Supplementary material for: What is the association between gender and self-perceived health status when controlling for disease-specific conditions? A retrospective data analysis of pre- and post-operative EQ-5D-5L differences in total hip and knee arthroplasty
Source: BMC Musculoskelet Disord. 2023 Nov 27;24:914. doi: 10.1186/s12891-023-07026-0 (PMC10680301; doi:10.1186/s12891-023-07026-0)
Supplement: Supplementary file 4 — Online resource 4. Descriptive statistics (full table). [file 12891_2023_7026_MOESM4_ESM.pdf]

Article title: What is the association between gender and self-perceived health status when controlling for disease-specific conditions? A retrospective data analysis of pre- and post-operative EQ-5D-5L differences in total hip and knee arthroplasty
Journal name: BMC Musculoskeletal Disorders
Author names: Anja Y. Bischof, Viktoria Steinbeck, David Kuklinski, Carlos J. Marques, Karina Bohlen, Karl C. Westphal, Frank Lampe, Alexander Geissler
Corresponding Author: Anja Y. Bischof, M.A., University of St. Gallen, School of Medicine, Chair of Health Care Management, St. Jakob-Strasse 21, 9000 St. Gallen, Switzerland, anja.bischof@unisg.ch

Online Resource 4 Descriptive statistics (full table)

**Independent-samples t-test**					
		**THA**		**TKA**	
**Variable**	**Sex**	**n**	**Mean (SD)**	**n**	**Mean (SD)**
Age (years)	Male	978	68.719 (9.045)	715	71.004[a] (7.902)
	Female	1390	68.862 (9.179)	915	70.002[a] (8.704)
LOS (days)	Male	978	7.338 (4.157)	715	7.438 (3.548)
	Female	1390	7.463 (4.014)	915	7.371 (3.418)
Surgery duration (min.)	Male	978	46.474 (18.060)	715	57.834[b] (17.669)
	Female	1390	45.756 (17.048)	915	55.256[b] (18.715)
In-hospital complication ratio (general)	Male	978	0.025 (0.155)	715	0.025 (0.157)
	Female	1390	0.019 (0.136)	915	0.015 (0.123)
In-hospital complication ratio (specific)	Male	978	0.009 (0.096)	715	0.015[a] (0.123)
	Female	1390	0.012 (0.110)	915	0.004[a] (0.066)
EQ-5D-5L (pre-surgery)	Male	978	0.576*** (0.275)	715	0.625*** (0.253)
	Female	1390	0.512*** (0.291)	915	0.534*** (0.284)
EQ-5D-5L (3-month FU)	Male	978	0.883** (0.156)	715	0.844*** (0.163)
	Female	1390	0.867** (0.172)	915	0.817*** (0.182)
EQ-5D-5L (12-month FU)	Male	978	0.910 (0.149)	715	0.885*** (0.154)
	Female	1390	0.903 (0.160)	915	0.862*** (0.169)
WOMAC (pre-surgery)	Male	895	45.658*** (15.516)	631	41.765*** (15.279)
	Female	1265	50.154*** (14.609)	837	46.435*** (14.392)
WOMAC (3-month FU)	Male	868	13.048 (12.189)	634	17.869*** (13.450)
	Female	1147	14.360 (12.594)	776	19.999*** (14.277)
WOMAC (12-month FU)	Male	852	11.016 (12.977)	609	14.430*** (13.640)
	Female	1144	11.978 (13.886)	704	17.543*** (15.127)
Elixhauser Comorbidity Score	Male	959	3.803** (4.957)	703	3.459 (4.773)
	Female	1373	3.314** (4.548)	907	3.261 (4.337)

**Mann-Whitney U test**						
	**THA**			**TKA**		
**Variable**	**Mean (SD)**	**Median**	**Min/ Max**	**Mean (SD)**	**Median**	**Min/ Max**

Pain	1.760*** (0.430)	2	0/2	1.692*** (0.465)	2	0/2
**Modified Kellgren-Lawrence Classification**						
Osteophytes	1.303 (0.481)	1	0/2	0.990 (0.099)	1	0/1
Joint space	2.340*** (0.540)	2	0/3	1.817 (0.393)	2	0/2
Sclerosis	2.329*** (0.644)	2	0/3	2.053 (0.401)	2	1/3
Deformation	1.159 (0.468)	1	0/2	0.998 (0.423)	1	0/2
**Severity of joint destruction in rheumatic diseases**						
Rheumatic disease with manifestation at the affected joint	0.032*** (0.175)	0	0/1	0.026*** (0.160)	0	0/1
Erosive joint destruction (according to Larsen-Dale-Eek)	0.986** (1.451)	0	0/4	2.860 (0.861)	3	1/5
**Pre-operative findings**						
Gonarthrosis				1.080** (0.278)	1	0/2
Malposition of the knee				0.679*** (0.867)	0	0/2
ASA classification	2.059*** (0.511)	2	1/4	2.140*** (0.474)	2	1/4
Post-operative specific complications	0.011 (0.104)	0	0/1	0.009** (0.096)	0	0/1
Post-operative generic complications	0.021 (0.144)	0	0/1	0.020 (0.139)	0	0/1
Walking distance at admission	2.101** (0.570)	2	1/5	2.101** (0.558)	2	1/5
Walking aid at admission	0.239*** (0.497)	0	0/3	0.277** (0.514)	0	0/3
Walking distance at discharge	2.321 (0.484)	0	1/4	2.355 (0.493)	2	1/4
Walking aid at discharge	1.004 (0.154)	2	0/2	1.012*** (0.162)	1	0/2
Patient Clincial Complexity Level	0.741 (1.103)	0	0/5	0.754 (1.094)	0	0/4
Early mobilization	0.770** (0.421)	1	0/1	0.706 (0.456)	1	0/1

Clinic type (general vs. specialized)	0.153** (0.360)	0	0/1	0.194 (0.396)	0	0/1

Statistically significant difference between sex at a **95% and ***99% significance level; SD= Standard Deviation; LOS = Length of stay; ASA= American Society of Anesthesiologists score.
