# Supplementary material for: What is the association between gender and self-perceived health status when controlling for disease-specific conditions? A retrospective data analysis of pre- and post-operative EQ-5D-5L differences in total hip and knee arthroplasty
Source: BMC Musculoskelet Disord. 2023 Nov 27;24:914. doi: 10.1186/s12891-023-07026-0 (PMC10680301; doi:10.1186/s12891-023-07026-0)
Supplement: Supplementary file 5 — Online resource 5. Full regression table for total hip arthroplasty (THA). [file 12891_2023_7026_MOESM5_ESM.pdf]

Article title: What is the association between gender and self-perceived health status when controlling for disease-specific conditions? A retrospective data analysis of pre- and post-operative EQ-5D-5L differences in total hip and knee arthroplasty
Journal name: BMC Musculoskeletal Disorders
Author names: Anja Y. Bischof, Viktoria Steinbeck, David Kuklinski, Carlos J. Marques, Karina Bohlen, Karl C. Westphal, Frank Lampe, Alexander Geissler
Corresponding Author: Anja Y. Bischof, M.A., University of St. Gallen, School of Medicine, Chair of Health Care Management, St. Jakob-Strasse 21, 9000 St. Gallen, Switzerland, anja.bischof@unisg.ch

Online Resource 5 Full regression table for total hip arthroplasty (THA)

THA	**mobility**			**self-care**			**usual activity**			**pain/discomfort**			**anxiety/depression**		
	pre	FU03	FU12	pre	FU03	FU12	pre	FU03	FU12	pre	FU03	FU12	pre	FU03	FU12
N=2368 (m=978; f=1390)	OR (CI)	OR (CI)	OR (CI)	OR (CI)	OR (CI)	OR (CI)	OR (CI)	OR (CI)	OR (CI)	OR (CI)	OR (CI)	OR (CI)	OR (CI)	OR (CI)	OR (CI)
**Age**	1.016 (1.005-1.027)	1.026 (1.011-1.042)	1.018 (1.002-1.034)	1.015 (1.001-1.029)	.994 (.967-1.021)	1.033 (1.004-1.063)	0.988 (0.978-0.998)	0.991 (0.976-1.007)	1.037 (1.007-1.067)	1.009 (0.998-1.020)	0.999 (0.987-1.012)	0.989 (0.976-1.003)	0.982 (0.972-0.993)	0.989 (0.969-1.009)	0.996 (0.977-1.015)
**WOMAC score** (at corresponding time)	1.052 (1.045-1.059)	1.129 (1.117-1.142)	1.132 (1.120-1.145)	1.062 (1.052-1.072)	1.108 (1.091-1.125)	1.113 (1.097-1.130)	1.057 (1.050-1.064)	1.135 (1.121-1.148)	1.113 (1.097-1.129)	1.088 (1.079-1.097)	1.155 (1.141-1.169)	1.147 (1.134-1.160)	1.028 (1.021-1.035)	1.079 (1.066-1.092)	1.077 (1.066-1.088)
**Elixhauser Comorbidity Index**	1.020 (1.000-1.040)	1.019 (.991-1.048)	1.049 (1.020-1.079)	1.010 (0.986-1.035)	1.015 (.965-1.067)	0.997 (0.951-1.046)	1.018 (0.999-1.038)	1.003 (0.974-1.033)	0.995 (0.949-1.043)	1.035 (1.014-1.056)	1.014 (0.989-1.039)	1.031 (1.005-1.058)	1.014 (0.994-1.035)	1.014 (0.976-1.054)	1.048 (1.013-1.085)
**Length of stay**		1.017 (.976-1.060)	1.013 (.977-1.051)		1.030 (.970-1.093)	1.042 (0.991-1.096)		1.059 (1.017-1.104)	1.033 (0.983-1.085)		1.002 (0.964-1.041)	0.970 (0.935-1.006)		1.051 (1.000-1.105)	1.027 (0.985-1.071)
**Surgery duration**		1.018 (1.008-1.028)	1.009 (.999-1.019)		1.005 (.987-1.023)	1.002 (0.984-1.021)		1.009 (0.999-1.020)	1.002 (0.985-1.020)		1.001 (0.992-1.010)	1.001 (0.992-1.010)		1.003 (0.989-1.018)	0.989 (0.975-1.002)
**Gender** (male vs female)	0.996 (0.829-1.196)	1.331 (1.032-1.718)	1.660 (1.275-2.162)	1.762 (1.392-2.231)	1.618 (1.015-2.577)	2.465 (1.519-4.003)	0.964 (0.806-1.153)	1.130 (0.858-1.488)	3.063 (1.890-4.964)	0.849 (0.698-1.032)	0.853 (0.682-1.067)	1.102 (0.875-1.387)	0.489 (0.401-0.598)	0.670 (0.458-0.979)	0.881 (0.625-1.243)
**ASA**															
1 vs 3	0.701 (0.476-1.031)	1.276 (.727-2.240)	.974 (.523-1.812)	0.331 (0.184-0.593)	1.887 (.637-5.590)	2.407 (0.869-6.664)	0.606 (0.416-0.882)	0.853 (0.473-1.538)	1.842 (0.687-4.938)	0.872 (0.579-1.313)	0.911 (0.561-1.479)	1.155 (0.700-1.906)	0.387 (0.254-0.591)	0.642 (0.279-1.480)	0.944 (0.433-2.060)

THA	mobility			self-care			usual activity			pain/discomfort			anxiety/depression		
	pre	FU03	FU12	pre	FU03	FU12	pre	FU03	FU12	pre	FU03	FU12	pre	FU03	FU12
2 vs 3	0.627 (0.483-0.813)	1.152 (.811-1.636)	.974 (.683-1.390)	0.597 (0.449-0.795)	1.337 (.739-2.417)	0.681 (0.388-1.194)	0.540 (0.421-0.692)	0.726 (0.503-1.047)	0.659 (0.379-1.145)	0.746 (0.576-0.968)	0.959 (0.692-1.328)	0.912 (0.653-1.274)	0.587 (0.453-0.760)	0.831 (0.517-1.336)	0.956 (0.619-1.474)
**Clinic type** (general vs specialized)		1.575 (0.982-2.527)	1.308 (0.812-2.109)		0.550 (0.251-1.204)	1.180 (0.521-2.673)		1.421 (0.863-2.340)	1.362 (0.610-3.038)		1.120 (0.747-1.681)	1.207 (0.794-1.835)		1.005 (0.529-1.909)	0.581 (0.327-1.031)
**Pain** (none or stress pain vs rest pain)	1.048 (0.846-1.299)	1.177 (0.873-1.585)	1.055 (0.768-1.449)	0.964 (0.717-1.297)	1.499 (0.844-2.661)	0.701 (0.365-1.348)	0.933 (0.754-1.154)	0.742 (0.528-1.042)	0.670 (0.349-1.286)	0.960 (0.760-1.213)	0.996 (0.768-1.292)	0.879 (0.667-1.157)	0.967 (0.766-1.221)	1,074 (0.695-1.659)	0.954 (0.626-1.452)
**Joint space**															
none or clearly narrowed vs lifted	1,188 (0.703-2.007)	0.858 (0.431-1.708)	1.183 (0.586-2.388)	1.027 (0.529-1.991)	0.253 (0.030-2.156)	1.437 (0.360-5.742)	1.480 (0.896-2.446)	1.340 (0.671-2.677)	1.306 (0.341-4.995)	1.841 (1.085-3.125)	1.250 (0.685-2.283)	1.274 (0.681-2.381)	0.982 (0.557-1.730)	1.432 (0.587-3.495)	0.880 (0.337-2.298)
advanced narrowed vs lifted	0.826 (0.669-1.020)	1.136 (0.837-1.542)	1.073 (0.784-1.469)	0.978 (0.741-1.290)	1.174 (0.665-2.072)	1.211 (0.686-2.136)	1.020 (0.829-1.254)	1.123 (0.808-1.560)	1.104 (0.633-1.928)	1.099 (0.877-1.376)	1.009 (0.776-1.312)	1.151 (0.876-1.512)	1.272 (1.016-1.592)	1.206 (0.784-1.854)	0.947 (0.637-1.408)
**Sclerosis**															
none or light sclerosis vs sclerosis with cysts	1.414 (1.020-1.959)	1.091 (0.689-1.726)	1.195 (0.750-1.906)	1.272 (0.843-1.919)	1.234 (0.573-2.657)	1.140 (0.513-2.536)	1.436 (1.048-1.969)	1.062 (0.660-1.708)	1.110 (0.506-2.433)	1.686 (1.207-2.356)	1.004 (0.661-1.524)	1.386 (0.921-2.085)	0.804 (0.573-1.129)	1.024 (0.557-1.881)	1.811 (1.040-3.154)
light sclerosis with cyst formation vs sclerosis with cysts	1.180 (0.957-1.454)	0.861 (0.639-1.161)	1.077 (0.789-1.469)	1.074 (0.814-1.417)	0.645 (0.368-1.127)	0.620 (0.357-1.075)	1.196 (0.974-1.468)	0.681 (0.494-0.939)	0.710 (0.413-1.219)	1.103 (0.883-1.378)	1.161 (0.898-1.501)	1.304 (0.996-1.708)	0.772 (0.619-0.963)	0.801 (0.530-1.210)	1.343 (0.909-1.985)
**Deformation**															
none vs clear deformation	0.927 (0.579-1.484)	1.224 (0.617-2.427)	1.644 (0.867-3.118)	0.544 (0.271-1.089)	0.410 (0.098-1.712)	0.572 (0.172-1.906)	0.882 (0.554-1.404)	1.086 (0.511-2.307)	0.646 (0.206-2.026)	0.570 (0.340-0.958)	1.525 (0.836-2.782)	1.876 (1.041-3.382)	1.088 (0.670-1.769)	1.778 (0.817-3.872)	0.683 (0.295-1.582)
light deformation vs clear deformation	1.052 (0.830-1.332)	1.072 (0.755-1.522)	0.808 (0.569-1.147)	0.840 (0.628-1.123)	1.175 (0.636-2.167)	0.802 (0.440-1.459)	0.882 (0.701-1.109)	1.360 (0.934-1.980)	0.764 (0.424-1.374)	0.986 (0.771-1.262)	1.332 (0.980-1.810)	1.194 (0.869-1.640)	0.994 (0.777-1.272)	0.865 (0.537-1.394)	0.762 (0.496-1.172)

THA	mobility			self-care			usual activity			pain/discomfort			anxiety/depression		
	pre	FU03	FU12	pre	FU03	FU12	pre	FU03	FU12	pre	FU03	FU12	pre	FU03	FU12
**Rheutmatic disease with manifestation at the affected joint** (no vs yes)	0.928 (0.568-1.517)	0.758 (0.382-1.501)	1.274 (0.548-2.960)	1.186 (0.640-2.199)	0.528 (0.180-1.552)	1.472 (0.347-6.244)	1.287 (0.793-2.089)	0.498 (0.253-0.981)	1.654 (0.400-6.847)	0.969 (0.589-1.594)	1.103 (0.600-2.026)	1.033 (0.523-2.043)	0.934 (0.575-1.519)	1.049 (0.422-2.608)	0.990 (0.403-2.429)
**Walking distance at admission**															
infinite (>500m) vs mobile on station level (50) or less	0.657 (0.453-0.953)	1.204 (0.687-2.111)	0.543 (0.294-1.005)	0.963 (0.568-1.634)	0.702 (0.241-2.041)	0.606 (0.186-1.972)	0.708 (0.489-1.024)	0.800 (0.427-1.499)	0.642 (0.199-2.076)	0.836 (0.557-1.254)	0.690 (0.433-1.098)	0.466 (0.281-0.774)	1.387 (0.934-2.058)	1.486 (0.729-3.030)	0.993 (0.495-1.992)
walking at a stretch up to 500m possible vs mobile on station level (50) or less	1.068 (0.833-1.370)	1.432 (1.013-2.023)	0.959 (0.681-1.349)	1.138 (0.846-1.530)	0.858 (0.488-1.510)	0.916 (0.520-1.612)	0.997 (0.786-1.266)	1.302 (0.906-1.869)	0.971 (0.555-1.698)	1.014 (0.788-1.304)	0.872 (0.648-1.173)	0.821 (0.605-1.115)	1.188 (0.920-1.535)	1.059 (0.663-1.691)	0.953 (0.626-1.453)
**Walking aid at admission** (no vs yes)	0.634 (0.500-0.804)	1.089 (0.782-1.515)	0.966 (0.693-1.347)	0.696 (0.528-0.917)	0.824 (0.464-1.464)	0.896 (0.507-1.581)	0.637 (0.508-0.799)	1.085 (0.763-1.542)	0.847 (0.490-1.467)	0.839 (0.660-1.067)	1.580 (1.171-2.131)	1.200 (0.885-1.626)	0.751 (0.592-0.952)	1.302 (0.821-2.068)	0.949 (0.634-1.419)
**Early mobilization** (no vs yes)		0.966 (0.720-1.297)	0.958 (0.707-1.300)		1.070 (0.634-1.807)	0.847 (0.494-1.450)		1.062 (0.777-1.450)	0.978 (0.582-1.644)		1.079 (0.835-1.395)	1.045 (0.803-1.360)		0.906 (0.607-1.352)	1.066 (0.736-1.543)
**pre-operative EQ-5D corresponding dimension**															
1 vs ≥3		0.214 (0.139-0.329)	0.549 (0.353-0.854)		0.118 (0.057-0.244)	0.096 (0.045-0.202)		0.209 (0.136-0.319)	0.242 (0.117-0.503)		0.288 (0.103-0.810)	0.772 (0.332-1.795)		0.097 (0.055-0.169)	.099 (.059-.168)
2 vs ≥3		0.353 (0.245-0.510)	0.554 (0.373-0.824)		0.458 (0.218-0.962)	0.295 (0.135-0.642)		0.379 (0.259-0.556)	0.464 (0.256-0.840)		0.691 (0.547-0.873)	0.843 (0.663-1.072)		0.312 (0.183-0.532)	0.443 (0.268-0.734)

THA	mobility			self-care			usual activity			pain/discomfort			anxiety/depression		
	pre	FU03	FU12	pre	FU03	FU12	pre	FU03	FU12	pre	FU03	FU12	pre	FU03	FU12
**R-squared (McFadden)**	0.096	0.303	0.316	0.123	0.352	0.391	0.108	0.334	0.365	0.174	0.298	0.311	0.056	0.247	0.253

Statistically significant difference between sex at a 95% (light grey) and 99% (dark grey) significance level; OR = Odds ratio; CI = 95% confidence interval; ASA= American Society of Anesthesiologists score.
