# Supplementary material for: What is the association between gender and self-perceived health status when controlling for disease-specific conditions? A retrospective data analysis of pre- and post-operative EQ-5D-5L differences in total hip and knee arthroplasty
Source: BMC Musculoskelet Disord. 2023 Nov 27;24:914. doi: 10.1186/s12891-023-07026-0 (PMC10680301; doi:10.1186/s12891-023-07026-0)
Supplement: Supplementary file 6 — Online resource 6. Full regression table for total knee arthroplasty (TKA). [file 12891_2023_7026_MOESM6_ESM.pdf]

Article title: What is the association between gender and self-perceived health status when controlling for disease-specific conditions? A retrospective data analysis of pre- and post-operative EQ-5D-5L differences in total hip and knee arthroplasty
Journal name: BMC Musculoskeletal Disorders
Author names: Anja Y. Bischof, Viktoria Steinbeck, David Kuklinski, Carlos J. Marques, Karina Bohlen, Karl C. Westphal, Frank Lampe, Alexander Geissler
Corresponding Author: Anja Y. Bischof, M.A., University of St. Gallen, School of Medicine, Chair of Health Care Management, St. Jakob-Strasse 21, 9000 St. Gallen, Switzer-land, anja.bischof@unisg.ch

Online Resource 6 Full regression table for total knee arthroplasty (TKA)

TKA	**mobility**			**self-care**			**usual activity**			**pain/discomfort**			**anxiety/depression**		
	pre	FU03	FU12	pre	FU03	FU12	pre	FU03	FU12	pre	FU03	FU12	pre	FU03	FU12
N=1629 (m=715; f=914)	OR (CI)	OR (CI)	OR (CI)	OR (CI)	OR (CI)	OR (CI)	OR (CI)	OR (CI)	OR (CI)	OR (CI)	OR (CI)	OR (CI)	OR (CI)	OR (CI)	OR (CI)
**Age**	1.014 (1.001-1.028)	0.989 (0.972-1.005)	1.003 (0.986-1.02)	1.034 (1.011-1.057)	1.015 (0.983-1.048)	0.999 (0.971-1.027)	0.979 (0.966-0.992)	0.971 (0.954-0.987)	0.997 (0.98-1.014)	0.981 (0.967-0.995)	0.984 (0.968-0.999)	0.986 (0.971-1.001)	0.992 (0.978-1.006)	0.993 (0.972-1.015)	0.995 (0.973-1.016)
**WOMAC score (at corresponding time)**	1.053 (1.045-1.061)	1.121 (1.108-1.134)	1.131 (1.118-1.144)	1.062 (1.047-1.077)	1.125 (1.102-1.148)	1.109 (1.091-1.127)	1.059 (1.05-1.068)	1.124 (1.111-1.138)	1.124 (1.112-1.137)	1.082 (1.072-1.093)	1.149 (1.133-1.164)	1.153 (1.139-1.168)	1.029 (1.02-1.037)	1.08 (1.066-1.093)	1.068 (1.056-1.08)
**Elixhauser Comorbidity In-dex**	0.999 (0.973-1.025)	0.99 (0.957-1.023)	1.01 (0.976-1.045)	1.005 (0.966-1.045)	1.039 (0.976-1.107)	1.03 (0.973-1.089)	1.01 (0.984-1.037)	1.024 (0.99-1.06)	1.041 (1.006-1.077)	1.008 (0.98-1.037)	1.02 (0.989-1.052)	1.031 (1.001-1.063)	1.015 (0.986-1.044)	0.971 (0.929-1.015)	0.98 (0.938-1.025)
**Surgery duration**		1.003 (0.995-1.011)	1 (0.992-1.007)		1 (0.985-1.016)	0.985 (0.972-0.999)		0.998 (0.99-1.007)	0.999 (0.992-1.007)		0.997 (0.99-1.004)	0.994 (0.988-1.001)		0.991 (0.981-1.002)	1 (0.99-1.009)
**Gender (male vs female)**	0.906 (0.726-1.13)	1.132 (0.845-1.515)	1.331 (0.983-1.803)	1.556 (1.086-2.23)	2.817 (1.582-5.016)	2.727 (1.595-4.662)	0.853 (0.684-1.065)	1.167 (0.864-1.578)	0.814 (0.6-1.104)	0.612 (0.479-0.783)	0.721 (0.554-0.939)	0.776 (0.597-1.009)	0.347 (0.268-0.45)	0.893 (0.589-1.354)	1.265 (0.843-1.899)
**ASA**															
1 vs 3	0.426 (0.241-0.754)	0.921 (0.429-1.98)	1.058 (0.479-2.337)	0.117 (0.016-0.869)	3.019 (0.568-16.044)	0 (0-)	0.65 (0.365-1.158)	1.446 (0.69-3.029)	1.093 (0.494-2.417)	0.334 (0.173-0.645)	1.106 (0.572-2.138)	1.126 (0.578-2.192)	0.526 (0.257-1.077)	0.811 (0.275-2.395)	0.711 (0.196-2.585)
2 vs 3	0.616 (0.459-0.827)	0.811 (0.57-1.155)	0.664 (0.466-0.946)	0.523 (0.353-0.775)	1.098 (0.571-2.112)	0.745 (0.424-1.311)	0.710 (0.533-0.944)	0.925 (0.643-1.331)	0.663 (0.466-0.943)	0.584 (0.43-0.793)	0.875 (0.623-1.228)	0.870 (0.626-1.208)	0.999 (0.723-1.38)	0.587 (0.371-0.93)	0.737 (0.467-1.165)

TKA	mobility			self-care			usual activity			pain/discomfort			anxiety/depression		
	pre	FU03	FU12	pre	FU03	FU12	pre	FU03	FU12	pre	FU03	FU12	pre	FU03	FU12
**Rheutmatic disease with manifestation at the affected joint** (no vs yes)	0.814 (0.428-1.549)	1.738 (0.699-4.32)	2.180 (0.838-5.671)	0.711 (0.289-1.754)	0.439 (0.106-1.815)	0.560 (0.155-2.017)	0.801 (0.425-1.508)	0.871 (0.377-2.015)	0.344 (0.167-0.711)	0.901 (0.456-1.781)	2.394 (1.051-5.455)	0.567 (0.277-1.158)	1.158 (0.582-2.303)	0.674 (0.244-1.86)	0.975 (0.342-2.774)
**Gonarthrosis** (primary vs secondary)	0.881 (0.592-1.311)	0.728 (0.439-1.207)	1.127 (0.659-1.927)	0.602 (0.335-1.082)	0.353 (0.152-0.816)	0.362 (0.175-0.751)	0.841 (0.567-1.247)	0.530 (0.322-0.873)	0.925 (0.55-1.558)	1.879 (1.203-2.937)	1.084 (0.682-1.724)	1.818 (1.134-2.915)	1.173 (0.741-1.857)	0.905 (0.442-1.852)	0.926 (0.476-1.804)
**Walking aid at admission** (no vs yes)	0.826 (0.641-1.064)	0.912 (0.664-1.252)	1.152 (0.825-1.609)	0.692 (0.476-1.007)	0.929 (0.511-1.688)	0.706 (0.419-1.191)	0.692 (0.54-0.888)	0.933 (0.675-1.29)	1.174 (0.843-1.635)	0.812 (0.621-1.062)	1.174 (0.877-1.572)	1.207 (0.899-1.62)	1.066 (0.81-1.403)	0.94 (0.619-1.426)	1.389 (0.892-2.162)
**Patient Clinical Complexity Level (PCCL)** (0vs1)	0.977 (0.769-1.241)	0.991 (0.729-1.346)	1.32 (0.958-1.821)	0.637 (0.435-0.933)	0.828 (0.458-1.495)	1.023 (0.595-1.758)	0.87 (0.686-1.103)	0.816 (0.597-1.115)	1.134 (0.825-1.56)	0.99 (0.765-1.28)	1.186 (0.900-1.562)	1.755 (1.327-2.321)	1.091 (0.84-1.417)	0.885 (0.592-1.323)	0.734 (0.489-1.102)
**Malposition of the knee**															
none vs severe varus knee		0.994 (0.713-1.385)	1.281 (0.907-1.809)		1.231 (0.631-2.402)	1.121 (0.623-2.017)		1.26 (0.893-1.778)	1.04 (0.741-1.461)		0.830 (0.62-1.111)	1.217 (0.91-1.627)		1.009 (0.644-1.58)	1.157 (0.735-1.821)
severe valgus knee vs severe varus knee		1.492 (0.949-2.347)	2.169 (1.367-3.439)		1.685 (0.702-4.045)	1.441 (0.656-3.166)		1.29 (0.798-2.085)	0.988 (0.617-1.581)		0.861 (0.569-1.301)	1.615 (1.074-2.427)		0.767 (0.414-1.422)	1.621 (0.898-2.923)
**Post-operative specific complications** (no vs yes)		0.206 (0.070-0.606)	0.326 (0.107-0.992)		1.105 (0.198-6.164)	1.822 (0.31-10.701)		0.912 (0.266-3.127)	0.395 (0.127-1.221)		0.314 (0.099-0.992)	0.823 (0.254-2.669)		1.341 (0.279-6.451)	0.335 (0.099-1.134)
**Walking distance at discharge**															
infinite (>500m) vs mobile on station level (50) or less		2.219 (0.458-10.746)	1.385 (0.242-7.923)		0 (0-0)	11.845 (1.063-131.953)		2.28 (0.427-12.163)	2.168 (0.39-12.058)		4.421 (0.802-24.381)	1.88 (0.443-7.97)		2.165 (0.23-20.384)	7.025 (1.278-38.63)
walking at a stretch up to 500m possible vs mobile on		0.838 (0.628-1.117)	1.073 (0.794-1.45)		1.318 (0.742-2.342)	1.864 (1.08-3.217)		1.356 (1.004-1.831)	1.16 (0.858-1.569)		1.253 (0.97-1.62)	0.974 (0.752-1.26)		0.888 (0.605-1.302)	1.093 (0.739-1.617)

TKA	mobility			self-care			usual activity			pain/discomfort			anxiety/depression		
	pre	FU03	FU12	pre	FU03	FU12	pre	FU03	FU12	pre	FU03	FU12	pre	FU03	FU12
station level (50) or less															
**Walking aid at discharge** (no vs yes)		0.845 (0.22-3.251)	0 (0-0)		0 (0-)	0 (0-)		1.672 (0.456-6.126)	0.476 (0.071-3.195)		2.035 (0.504-8.224)	1.087 (0.252-4.684)		1.703 (0.309-9.38)	0 (0-0)
**pre-operative EQ-5D corresponding dimension**															
1 vs ≥3		0.148 (0.09-0.243)	0.296 (0.18-0.485)		0.140 (0.053-0.368)	0.157 (0.067-0.370)		0.147 (0.090-0.239)	0.246 (0.153-0.396)		0.524 (0.252-1.088)	0.389 (0.169-0.892)		0.042 (0.022-0.08)	0.094 (0.049-0.18)
2 vs ≥3		0.272 (0.175-0.423)	0.447 (0.287-0.696)		1.032 (0.375-2.842)	1.147 (0.461-2.852)		0.321 (0.202-0.509)	0.426 (0.27-0.67)		0.615 (0.467-0.81)	0.664 (0.509-0.868)		0.242 (0.133-0.44)	0.457[b] (0.242-0.862)
**R-squared (McFadden)**	0.085	0.299	0.321	0.137	0.408	0.526	0.104	0.325	0.315	0.164	0.309	0.331	0.065	0.285	0.218

Statistically significant difference between sex at a 95% (light grey) and 99% (dark grey) significance level; OR = Odds ratio; CI = 95 % confidence interval; ASA= American Society of Anesthesiologists score.
